# Clinical and Etiological Exploration of Ventilator-Associated Pneumonia in the Intensive Care Unit of a Developing Country

**DOI:** 10.7759/cureus.47515

**Published:** 2023-10-23

**Authors:** Omar Nisar, Samaha Nisar, Shahbaz Khattak Haroon Ur Rashid, Syed Muhammad Ibne Ali Jaffari, Zaki Haider, Fiza Fatima, Shan e Zahra, Ali Hassan Ijaz, Mehwish Kaneez, Gulfam Khan Shairwani

**Affiliations:** 1 Internal Medicine, Shalamar Medical and Dental College, Lahore, PAK; 2 Internal Medicine, Shifa International Hospital Islamabad, Islamabad, PAK; 3 Internal Medicine, Rashid Latif Medical College, Lahore, PAK; 4 Internal Medicine, Faisalabad Medical University, Faisalabad, PAK; 5 Pediatric Oncology, Shaukat Khanum Memorial Cancer Hospital and Research Centre, Lahore, PAK; 6 Pediatrics, Rawalpindi Medical University, Rawalpindi, PAK; 7 Critical Care Medicine, District Headquarter Hospital, Rawalpindi, PAK

**Keywords:** developing country, etiological factors, nosocomial infections, intensive care unit, ventilator-associated pneumonia

## Abstract

Background

Ventilator-associated pneumonia (VAP) is a critical concern in the intensive care unit (ICU), with significant implications for patient outcomes. This retrospective cross-sectional study aimed to determine the prevalence of VAP in an ICU of a developing country, identify the predominant etiological factors, assess patient outcomes, and underscore the need for tailored interventions in high-risk patient groups.

Methods

This retrospective cross-sectional study included 589 ICU patients who underwent ventilator-assisted breathing for over 48 hours. Among them, 151 developed VAP. The diagnosis was made on clinical, laboratory, and radiological findings, and tracheal aspirate cultures. Exclusions included pediatric patients, less than 48 hours of ventilation, and pre-existing lung infections. Patient data encompassed gender, age, comorbidities, outcomes, admission reasons, isolated microorganisms, and clinical findings.

Results

151 patients out of the 589 developed VAP. The age of the patients ranged between 31 to 69 years and the mean age was 45.43 ± 8.92 years. Clinical diagnoses upon ICU admission varied, including sepsis, trauma, stroke, and metabolic disorders. Chest X-rays commonly revealed atelectasis (19.2%), consolidation (21.9%), pleural effusion (11.9%), and lobar pneumonia (45.7%). Tracheal aspirate cultures predominantly isolated multidrug-resistant gram-negative rods, with methicillin-resistant gram-positive cocci and fungal pneumonia prevalent in neutropenic sepsis cases. Notably, only 54 (35.8%) of patients survived, with significantly poorer outcomes observed in sepsis, neutropenic sepsis, and stroke cases compared to trauma and post-operative admissions.

Conclusion

Multidrug-resistant organisms and the spread of nosocomial infections are the predominant causes of VAP in the ICU. This emphasizes the urgent need for multifaceted interventions to prevent and manage VAP effectively. Developing and implementing targeted strategies, considering the unique challenges faced in resource-constrained healthcare settings can aid in decreasing the mortality associated with it.

## Introduction

Ventilator-associated pneumonia (VAP) is a grave nosocomial infection that significantly impacts patients in intensive care unit (ICU) [1]. It is a leading cause of morbidity and mortality among critically ill patients, often prolonging hospital stays and increasing healthcare costs [2]. The challenge posed by VAP is further increased in the context of developing countries, where resource limitations and unique patient demographics can influence the clinical and etiological landscape of this condition [3].

VAP is an infectious complication that manifests 48 hours after the commencement of mechanical ventilation. It is associated with a constellation of risk factors, including prolonged intubation, impaired host defense mechanisms, poor infection control measures, and the aspiration of oropharyngeal secretions [4]. These factors contribute to the colonization of the respiratory tract by pathogenic microorganisms, leading to pneumonia [2,3]. The clinical presentation of VAP encompasses various symptoms such as fever, purulent sputum production, and increased oxygen requirement on the ventilator [2,5]. However, the etiological microorganisms causing VAP vary geographically (in developed versus developing countries) due to changes in microbial resistance patterns, patient populations, and healthcare practices [3,6]. Gram-negative bacteria are among the most frequently isolated pathogens in VAP cases. Literature from developing countries shows that *Klebsiella pneumoniae* and *Pseudomonas aeruginosa* are the predominant culprits of VAP in ICU [6,7]. However, there is limited research focused specifically on the clinical and etiological spectrum of VAP within the ICU settings of developing countries.

While numerous studies have explored VAP in well-resourced healthcare systems, there is a lack of data addressing its magnitude in the context of developing countries. This knowledge gap is significant, as factors such as differing patient demographics, healthcare infrastructure, reason for ICU admission, and antimicrobial resistance patterns can profoundly influence the presentation and etiology of VAP [4-6]. A recent investigation from Pakistan highlighted that in a resource-limited setting, multidrug-resistant organisms were responsible for a substantial proportion of VAP cases, emphasizing the challenges posed by antimicrobial resistance in these regions [8]. Given the limited research on VAP in developing countries, this study aims to bridge the knowledge gap by examining the clinical and etiological spectrum of VAP among patients admitted to ICU in a developing country. Our research seeks to provide insights that can inform targeted prevention and treatment strategies by elucidating the unique characteristics of VAP within this context.

## Materials and methods

This retrospective cross-sectional study was conducted at the Department of Critical Care, District Headquarters Hospital, Rawalpindi, Pakistan from May 2018 till May 2022. The study included patients admitted to ICU after surgery, sepsis, trauma, stroke, and metabolic disorders. During the study period, a total of 589 patients underwent ventilator-assisted breathing for more than 48 hours, of which 151 patients developed VAP were defined as an infection in the trachea-bronchial tree in patients with endotracheal intubation (or inserted tracheostomy tube) and were ventilated for more than 48 hours. The diagnosis of VAP was made based on the patient's clinical condition (Fever > 38 °C or hypothermia < 36 °C), laboratory findings (leukocytosis or leukopenia, blood cultures, lactate, and C-reactive protein levels), radiological findings (new or intensifying infiltrates in chest X-ray), and results of tracheal aspirate cultures reported with antibiogram. Patients aged below 18 years, ventilation time less than 48 hours, and pre-existing lung infection were excluded from the study. All patients were treated with intravenous antibiotics according to the results of culture and sensitivity patterns. Ethical approval was obtained from the hospital's Institutional Review Board (IRB) of Rawalpindi Medical University (approval number 2018-M-015-RMU) before conducting the study.

Patients were studied for multiple clinical and demographical variables including gender, age, comorbidities, outcomes, reason for ICU admission, isolated microorganisms on tracheal aspirate, and reason for ICU admission. Patient files, reports, and computer records were utilized for data extraction. Categorical variables including comorbidities, the reason for admission, isolated organisms, outcomes, and clinical findings were expressed as frequencies and percentages. Numerical variables were expressed as means and standard deviation. Statistical Package for Social Sciences (IBM Corp., Armonk, NY, USA) version 25.0 was utilized for data entry, interpretation, and analysis.

## Results

Out of 589 patients, 151 (26.3%) patients developed VAP. The mean age of these patients was 45.43 ± 8.92 years, ranging from 31 to 69 years. Most of the patients had multiple co-morbidities. All patients with malignancy had a diagnosis of acute leukemia. The baseline demographic details of the study participants are shown in Table 1.

**Table 1:** Baseline demographical details of study participants. VAP: Ventilator-associated pneumonia

Parameter		Frequency	Percentage
Gender	Male	72	47.7%
	Female	79	52.3%
Smoking status	Smoker	58	38.4%
	Non-smoker	93	61.6%
Co-morbidities	Diabetes mellitus	49	32.5%
	Hypertension	57	37.7%
	Chronic heart disease	21	13.9%
	Malignancy	32	21.2%
	Liver cirrhosis	9	5.9%
	Obesity	19	12.6%
Time from intubation till diagnosis of VAP	48-72 hours	23	15.2%
	72-96 hours	48	31.8%
	96-120 hours	39	25.8%
	More than 120 hours	41	27.1%

Patients were presented with many different clinical diagnoses including non-neutropenic sepsis, post-operative monitoring, neutropenic sepsis, hepatic encephalopathy, and stroke. Moreover, atelectasis, consolidation, pleural effusion, and pulmonary infiltrates were common findings observed in chest radiography. Most of the patients had more than one finding on chest X-ray. These statistics are also tabulated in Table 2.

**Table 2:** A tabulation of reasons for ICU admissions and outcomes. ICU: Intensive Care Unit

Parameters		Frequency	Percentages
Reason for ICU admission	Non-neutropenic Sepsis	35	23.2%
	Trauma	22	14.6%
	Post-operative monitoring	30	19.9%
	Neutropenic sepsis	32	21.1%
	Cerebrovascular accident/stroke	29	19.2%
	Hepatic encephalopathy	3	2%
Findings on chest radiograph	Atelectasis	29	19.2%
	Consolidation	33	21.9%
	Lobar pneumonia	69	45.7%
	Pulmonary infiltrates	16	10.6%
	Pleural effusion	18	11.9%
	Cardiomegaly	4	2.6%
	Apical pneumothorax	3	2%
Mode of delivery of ventilation	Endotracheal tube	103	68.2%
	Tracheostomy tube	48	31.8%
Outcomes	Alive	54	35.8%
	Dead	97	64.2

The results from tracheal aspirate cultures elucidate that most isolated organisms were multidrug-resistant gram-negative rods. Furthermore, methicillin-resistant gram-positive cocci and fungal pneumonia were also predominantly seen in patients with neutropenic sepsis. These findings are further delineated in Table 3.

**Table 3:** A tabulation of isolated infectious organisms from tracheal aspirate cultures.

Organism type	Isolated organism	Frequency	Percentages
Gram-negative rods	Piperacillin/tazobactam sensitive *Pseudomonas aureginosa*	19	12.6%
	Meropenem sensitive *Pseudomonas aureginosa*	16	10.6%
	Multi-drug resistant *Pseudomonas aureginosa*	11	7.3%
	Multi-drug resistant *Acinetobacter baumannii*	19	12.6%
	Multi-drug resistant *Klebsiella pneumophilia*	20	13.2%
	Multi-drug resistant *Escherichia coli*	24	15.9%
	Multi-drug resistant *Stenotrophomonas maltophilia*	13	8.6%
Gram-positive Cocci	Methicillin-resistant *Staphylococcus aureus*	21	13.9%
	Vancomycin-resistant *Staphylococcus aureus*	2	1.3%
Fungal organisms	Blastomycosis	1	0.7%
	Invasive Aspergillosis	2	1.3%
	Fluconazole resistant *Candida glabrata*	3	2%

**Figure 1:**
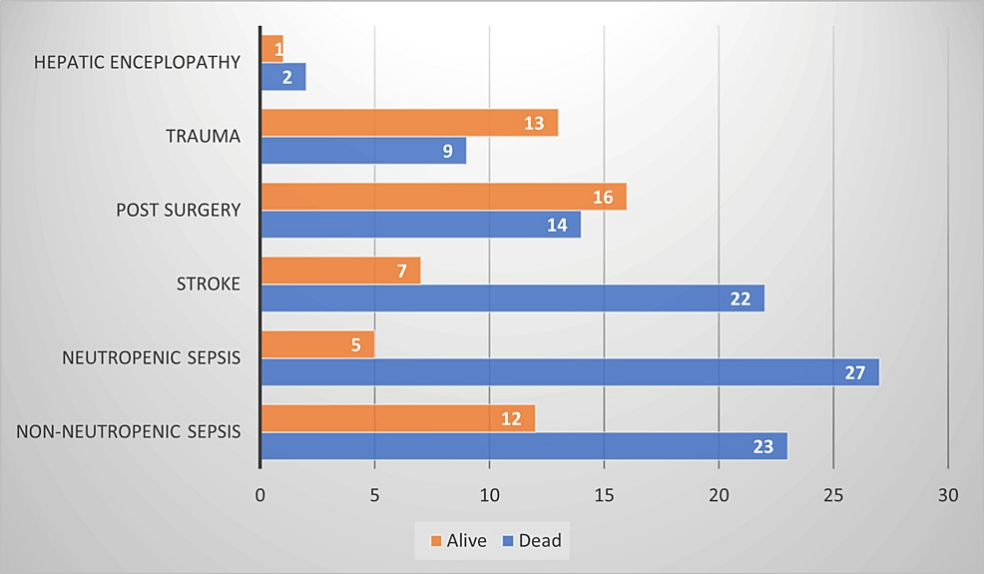
A depiction of outcomes of VAP across each diagnosis. VAP: Ventilator-associated pneumonia

Of the 151 patients who developed VAP, only 54 (35.8%) survived and were successfully treated and extubated. The remaining 97 (64.1%) patients succumbed to progressive pneumonia and expired. Patients with non-neutropenic sepsis, neutropenic sepsis, and stroke experienced poorer outcomes than patients admitted due to trauma, and post-surgery. The comparison of outcomes with each diagnosis is further delineated in Figure 1.

## Discussion

Our comprehensive examination of VAP in a developing country has yielded critical insights into the prevalence, etiological factors, and clinical outcomes associated with this formidable healthcare challenge. In our study, the prevalence of VAP among patients requiring prolonged mechanical ventilation was high at 26.3%. Other studies from developed countries have reported lower prevalence rates which report rates ranging between 5% to 14% [4,9,10]. The higher prevalence in our setup is due to poorer healthcare practices, a lack of resources, and suboptimal infection control measures in ventilated patients. This finding explains the magnitude of the problem and necessitates the need for tailored strategies and interventions to address this clinical challenge, particularly in resource-constrained settings.

Patients in our study presented with diverse clinical diagnoses upon ICU admission. These diagnoses included non-neutropenic sepsis, post-operative monitoring, neutropenic sepsis, hepatic encephalopathy, and stroke. Notably, the presence of VAP was not limited to a particular clinical subgroup, suggesting that VAP is a pervasive concern across various medical conditions and underscores the need for a broad-based approach to its prevention [11]. Chest radiography played a crucial role in our study, revealing common findings such as atelectasis, consolidation, pleural effusion, and pulmonary infiltrates. Most patients exhibited multiple findings on chest X-rays, indicating the complexity of respiratory issues after diagnosis of VAP. These findings were consistent with other studies as well and highlight the importance of serial radiological assessments in ventilated ICU patients to detect and manage complications promptly [12,13].

Microbiological analysis of tracheal aspirate cultures provided valuable insights into the etiology of VAP in our patient population. The prominence of multidrug-resistant gram-negative rods reflects the global trend in the increasing resistance of pathogens to antibiotics [14]. A study from India reported a predominance of multi-drug resistant gram-negative rods especially *Acinetobacter* species in their study which is also consistent with our study findings [15]. As reported in other studies, multi-drug resistant organisms causing VAP pose significant challenges to healthcare costs, prolonged ICU stays, and risk of mortality particularly in resource-limited countries [2,16]. This underscores the urgent need for antimicrobial stewardship programs and infection control measures to mitigate the spread of drug-resistant organisms in such healthcare settings [14,17]. Furthermore, our study identified the prevalence of methicillin-resistant gram-positive cocci and fungal organisms, particularly among patients with neutropenic sepsis. These immunocompromised patients are at heightened risk for VAP caused by the invasion of opportunistic pathogens [18]. Furthermore, the abundance of *E. coli* strains causing VAP in our population is also of huge concern. *E. coli*-associated VAP shows poor infection control measures and poor hand hygiene measures by healthcare practitioners [19]. Other studies from developing countries show similar results with an increased incidence of *E.coli*-associated VAP due to poor infection control measures [16,19]. Tailored interventions, such as targeted prophylaxis and stringent infection control practices, must be warranted to control these infections in developing countries. [9,18].

Assessment of outcomes of VAP was also a critical aspect of our study. Our study yielded a survival rate of 35.8% which is consistent with another Indian study that reports survival of nearly 32% [15]. Notably, patients with neutropenic sepsis and stroke had higher mortality rates than the rest of the study cohort. This disparity in outcomes may reflect the underlying severity of the patient's conditions upon ICU admission and necessitates appropriate steps for prevention strategies to prevent nosocomial-associated healthcare infection in these high-risk groups [11,14,18].

In recent years, there have been significant advances in the prevention of VAP in ICU settings. Healthcare providers now emphasize stringent hand hygiene and proper infection control measures, including the use of sterile gloves and gowns during intubation [4,12]. Additionally, there's a growing focus on elevating the head of the patient's bed at a 30-45-degree angle to reduce the risk of aspiration [8,9]. Advanced ventilator technologies with features like subglottic secretion suctioning and closed suction systems have also been developed to minimize the risk of infection [13]. Furthermore, comprehensive oral care protocols and selective decontamination of the digestive tract are increasingly employed to prevent VAP, resulting in improved patient outcomes and reduced healthcare costs.

Several limitations must be acknowledged when interpreting the study findings. The retrospective nature of the study and reliance on medical records may introduce selection bias and limit the availability of certain data points. Additionally, our study was conducted in a single healthcare facility in a developing country, which may limit the generalizability of our findings to other settings. Nonetheless, our research underscores the importance of identifying high-risk patients and implementing tailored interventions for this vulnerable group. Moreover, the study is a crucial step in advancing our knowledge of VAP in developing countries, providing a basis for future research and interventions aimed at alleviating this burden and enhancing patient care in similar healthcare settings. Collaborative endeavors involving healthcare providers, policymakers, and researchers are essential to effectively address the multifaceted challenges posed by VAP.

## Conclusions

The prevalence of VAP in an ICU setting in a developing country is alarming. With over a quarter of mechanically ventilated patients affected, urgent measures are warranted to reduce its incidence. The dominance of nosocomial multidrug-resistant gram-negative bacilli underscores the need for rigorous infection control and antimicrobial stewardship in developing countries. Additionally, the mortality rate among VAP patients, particularly in cases of sepsis, neutropenic sepsis, and stroke, emphasizes the need for appropriate steps to mitigate the crisis. Although our study has limitations, including its retrospective design, its findings underscore the importance of tailored interventions to reduce the impact of VAP in resource-constrained settings. Collaborative efforts involving healthcare providers, policymakers, and researchers are essential to develop effective strategies to improve care in critically ill ventilated patients.
